# Low Platelet to White Blood Cell Ratio Indicates Poor Prognosis for Acute-On-Chronic Liver Failure

**DOI:** 10.1155/2018/7394904

**Published:** 2018-05-08

**Authors:** Yusheng Jie, Jiao Gong, Cuicui Xiao, Shuguang Zhu, Wenying Zhou, Juan Luo, Yutian Chong, Bo Hu

**Affiliations:** ^1^Department of Infectious Diseases, Key Laboratory of Liver Disease of Guangdong Province, Third Affiliated Hospital of Sun Yat-sen University, Guangzhou, China; ^2^Department of Laboratory Medicine, Third Affiliated Hospital, Sun Yat-sen University, Guangzhou, China; ^3^Cell-Gene Therapy Translational Medicine Research Center, Key Laboratory of Liver Disease of Guangdong Province, Third Affiliated Hospital of Sun Yat-sen University, Guangzhou, China; ^4^Department of Hepatic Surgery, Third Affiliated Hospital of Sun Yat-sen University, Guangzhou, China

## Abstract

*Background. *Platelet to white blood cell ratio (PWR) was an independent prognostic predictor for outcomes in some diseases. However, the prognostic role of PWR is still unclear in patients with hepatitis B related acute-on-chronic liver failure (ACLF). In this study, we evaluated the clinical performances of PWR in predicting prognosis in HBV-related ACLF.* Methods. *A total of 530 subjects were recruited, including 97 healthy controls and 433 with HBV-related ACLF. Liver function, prothrombin time activity (PTA), international normalized ratio (INR), HBV DNA measurement, and routine hematological testing were performed at admission.* Results*. At baseline, PWR in patients with HBV-related ACLF (14.03 ± 7.17) was significantly decreased compared to those in healthy controls (39.16 ± 9.80). Reduced PWR values were clinically associated with the severity of liver disease and the increased mortality rate. Furthermore, PWR may be an inexpensive, easily accessible, and significant independent prognostic index for mortality on multivariate analysis (HR = 0.660, 95% CI: 0.438–0.996, *p* = 0.048) as well as model for end-stage liver disease (MELD) score.* Conclusions*. The PWR values were markedly decreased in ACLF patients compared with healthy controls and associated with severe liver disease. Moreover, PWR was an independent prognostic indicator for the mortality rate in patients with ACLF. This investigation highlights that PWR comprised a useful biomarker for prediction of liver severity.

## 1. Introduction

Acute-on-chronic liver failure (ACLF) is a critical clinical syndrome involving acute hepatic insult manifesting as jaundice and coagulopathy, complicated within 4 weeks by ascites and/or encephalopathy in a patient with previously diagnosed or undiagnosed chronic liver disease [1, 2]. Approximately 350 million persons are chronically infected with hepatitis B (HBV) in the world. Chronic HBV infection is the major cause of ACLF in China. ACLF has a high short-term mortality which exerts a heavy economic toll on patients and society [3, 4]. The model of end-stage liver disease (MELD) score is developed to classify patients with cirrhosis awaiting transplantation according to the severity of their liver disease and is based on variables related to both liver and renal function. Increasing MELD score was reported to be associated with increasing severity of hepatic dysfunction and increased three-month mortality risk in patients. However, the widely used MELD score could not take into account the inflammation.

Platelet to white blood cell ratio (PLT/WBC) refers to a hematologic marker of the systemic inflammatory response. Recently, PWR has been validated not only to forecast risk of infectious complications in patients undergoing radical nephrectomy for renal malignancy, but also to predict outcomes in ischemic stroke patients with intravenous thrombolysis [5, 6]. In addition, PWR is a reliable infectious indicator that even could differentiate between infection and the normal response after splenectomy for trauma [7]. Patients with ACLF had a significantly higher white cell count and low platelet count. However, whether PWR, a combination of platelet and white blood cell, might prognosticate survival of HBV-related ACLF is not explored. We also investigated the association between the severity of liver and PWR in ACLF patients.

## 2. Materials and Methods

### 2.1. Subjects

We retrospectively reviewed the demographic and baseline characteristics of patients with ACLF who were admitted to Department of Infectious Disease, Third Affiliated Hospital of Sun Yat-sen University in China, between February 1, 2013, and June 1, 2015. Several patients with ACLF have been reported [8]. The inclusion criteria of ACLF were (1) the presence of HBsAg in the serum for at least 6 months; (2) patient with the history of chronic hepatitis or cirrhosis (chronic hepatitis was diagnosed by persistent or intermittent elevation in AST or ALT levels. Cirrhosis was diagnosed by liver biopsy or supported by imaging technologies (hepatic ultrasound and/or CT, MRI)); (3) acute hepatic insult manifesting as jaundice (TBIL ≥ 85 *μ*mol/L) and coagulopathy (international normalized ratio (INR) ≥ 1.5 or prothrombin activity < 40%), complicated within 4 weeks by ascites and/or encephalopathy; and (4) evidence of active viral replication, as documented by measurable HBV DNA in the serum (≥2.0 log IU/ml). A patient was excluded (1) if he or she was coinfected with HAV, HCV, HDV, HEV or HIV, (2) if he or she had drug-induced hepatitis or alcoholic hepatitis, (3) if he or she was diagnosed with fatty liver disease or Wilson disease, (4) if he or she had received steroids 6 months before admission, and (5) if he or she had malignant jaundice induced by obstructive jaundice or hemolytic jaundice and prolonged prothrombin time induced by blood system disease. Blood samples were collected from all HBV-infected patients within 24 hours after admission, and blood samples were taken from 97 healthy individuals at the time of recruitment. Neither the technicians who did the laboratory tests nor the investigators who followed the patients knew the diagnosis of these patients. Patients received antiviral therapy and supportive treatment after hospitalization.

The study was approved by the institutional ethics committee of the Third Affiliated Hospital of Sun Yat-sen University.

### 2.2. Laboratory Methods

White blood cell count (WBC), red blood cell count (RBC), hemoglobin level (HB), and platelets (PLT) were determined using the XE-5000 automated hematology analyzer, as one part of a complete blood cell count. Venous blood samples were collected at admission and the following tests were performed: complete blood count, renal (creatinine), liver (ALT, AST, GGT, albumin, and total bilirubin), and the AFP, HBV DNA. All biochemical parameters were measured by standard automated laboratory methods and using commercially available kits according to the manufacturers protocols. The PTA and INR were evaluated by the STAGO full automatic analyzer. At baseline, demographic and clinical characteristics, including the model for end-stage liver disease (MELD) score (with higher scores indicating more severe illness), were collected.

### 2.3. MELD Score

Liver disease severity at admission was evaluated via the MELD score using a mathematical formula that is based on three lab results: total bilirubin; creatinine levels; and the INR for prothrombin time to predict survival. The MELD score was calculated using the website calculator (https://www.mayoclinic.org/gi-rst/mayomodel7.html).

### 2.4. Statistical Analysis

Data were expressed as the median ± standard error of the mean (SEM), unless indicated otherwise. We used the Student's *t*-test and the Mann–Whitney *U* test for analyses that compared two groups. Continuous variables were compared using the *t*-test or Mann–Whitney *U* test for variables with an abnormal distribution. Survival curves were depicted using the Kaplan-Meier method and compared using the log-rank test. Cox regression analysis was used for multivariate analyses. Categorical variables were compared by Chi-square test or Fisher's exact probability test. We used SPSS version 16.0 (SPSS, Inc., Chicago, IL) to perform statistical procedures. A value of *p* < 0.05 was considered statistically significant.

## 3. Results

### 3.1. Increased PWR Values in Patients with ACLF

A total of 530 subjects, including 433 with HBV-related ACLF and 97 healthy controls, were enrolled in this study (Table 1). The control group consisted of 72 males and 25 females (*n* = 97). The ratio of male to female in the ACLF group was 360/73 and no significant difference was observed among the two groups (*p* = 0.059). INR, ALT, AST, and total bilirubin were significantly increased, and albumin was markedly reduced in patients with ACLF compared with those in healthy control (Table 1). The mean PWR value in the HBV-related ACLF patients was 15.03 ± 7.17, with a median of 14.03 (range, 1.14 to 42.86). The PWR in patients with ACLF (14.03 ± 7.17) was significantly lower than those in healthy controls (39.16 ± 9.80) (*p* < 0.001).

### 3.2. Baseline Characteristics and Baseline Factors Related to the PWR Value

Serum PTA and TBIL are indicators of liver functions. Other than a moderate negative correlation between total bilirubin and PWR (*r* = −0.1485, *p* < 0.001), there was a moderate positive correlation between PWR and RBC (*r* = 0.2348, *p* < 0.001), HB (*r* = 0.1887, *p* < 0.001) in ACLF patients (Table 2). We found that PWR was negatively correlated with MELD score (*r* = −0.1410, *p* = 0.0032), implying the important role of PWR in prediction for the severity of disease.

### 3.3. Association of PWR with 3-Month Mortality in HBV-Infected Patients

**Figure 1:**
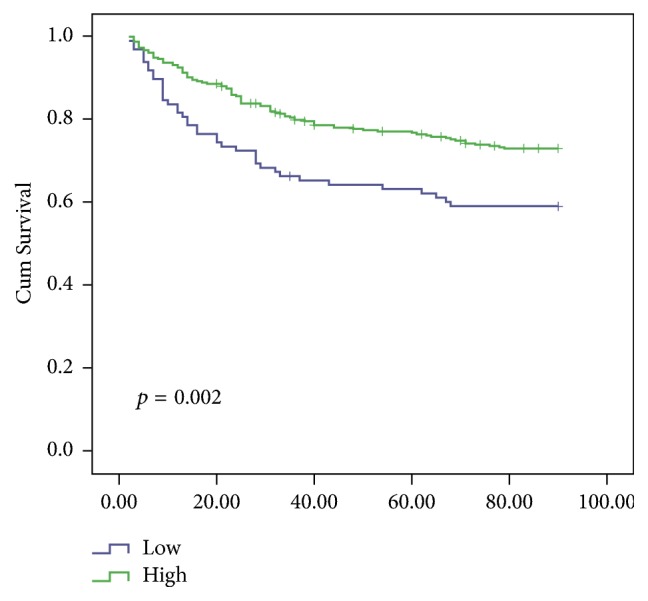
Kaplan-Meier survival curves of the ACLF patients categorized by the PWR value. The curves showed different survival rates of patients with different PWR value.

**Figure 2:**
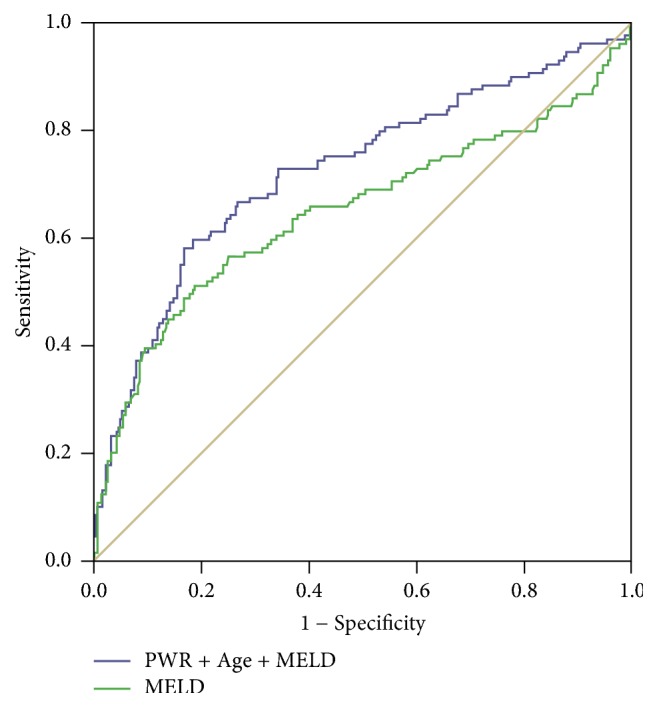
ROC curve analysis for prediction of mortality by MELD scores and the combination of the PWR values, age, and MELD score. The MELD scores (green line) and the combination (blue line) were indicated.

The median follow-up was 90 days (range, 2–207 days). During the follow-up, 129 patients with ACLF died. The mortality rate was significantly higher in patients with low PWR value than in patients with high PWR value (Figure 1) (*p* = 0.002). And, the mortality rate was significantly higher in patients with high MELD score than in patients with low MELD score (*p* < 0.001) (S Figure 1). The multivariate logistic regression analysis had shown that PWR, age, and MELD score were independent factors predicting mortality rate (Table 3). Hence, PWR, age, and MELD score were combined to predict mortality and the AUC was 0.725 ± 0.029 (*p* < 0.001) based on the receiver operating characteristic curves (ROC) analysis (Figure 2). The AUC values were 0.649 ± 0.033 for the MELD score alone.

The “minimum *p* value” approach was applied to estimate an optimal cut-off value for PWR using X-tile software and 433 patients were stratified into low PWR (PWR < 9.0) and high PWR (PWR ⩾ 9.0) groups according to the data obtained from complete blood count. Differences in clinical and laboratory characteristics are listed in Table 4 among the two groups of PWR. The MELD score in low PWR group is higher than that in high group. Furthermore, patients with lower values of PWR had higher levels of age, WBC, TBIL, creatinine, and mortality and had lower levels of PLT and HB. The serum INR and ascites were not significantly different among the two groups.

## 4. Discussion

PWR calculated from complete blood count is very inexpensive and easy to obtain. In the current study, HBV-related ACLF patients had significantly lower PWR values than healthy subjects. Importantly, decreasing PWR values may be used as an independent predictor of mortality in HBV-related ACLF patients.

The mechanisms underlying the correlation between PWR and severity of hepatitis B are unclear. Some reasons might account for the decreased PWR level in ACLF patients. Decreased peripheral platelet count was associated with liver fibrosis in patients with chronic hepatitis B [9, 10]. Other than hypersplenism, increased destruction of platelets, decreased platelet production, antiplatelet antibodies, disseminated intravascular coagulation, translocated toxins, and other gut-derived substances might cause low platelet counts [11, 12]. In addition, bacterial infections were the most common causes of a high white blood cell count. As a result, PWR might be decreased in patients with HBV infection. Previous studies also had shown that PWR was a prognostic predictor for 90-day outcomes in ischemic stroke patients with intravenous thrombolysis [6]. Moreover, our study had suggested that there was a correlation between PWR and the severity of liver disease. Patients with lower values of PWR tended to be older and had higher total bilirubin, MELD score, and mortality rate.

Our study suggested that decreasing PWR values could serve as an independent predictor of mortality in HBV-infected patients. The model of end-stage liver disease (MELD) score, including variables related to both liver and renal function, was used widely to classify patients with cirrhosis awaiting transplantation according to the severity of their liver disease [13]. However, the PWR was based on only two markers, which make it simpler and easier to calculate than the MELD score. In the present study, we reported that the combination of the PWR value with the MELD score and age could be used for predicting HBV-infected patients' mortality, which had a higher prediction power (AUC = 0.725 ± 0.029, *p* < 0.001) than that of MELD score (AUC = 0.649 ± 0.033, *p* < 0.001). The present study included 94 healthy controls and 433 patients with HBV-related ACLF, and the mortality rate was significantly high in patients with ACLF. In this study, we found that 129 patients with ACLF died before the 3-month follow-up period. PWR could predict the mortality in patients with hepatitis B.

PWR could be a valuable and reliable tool of disease activity in patients with HBV-related ACLF. Limitation of this study includes its retrospective analysis with potential biases such as selection bias. Despite having this limitation, our investigation generates important findings. In other words, this is the first study to investigate the effectiveness of PWR in patients with HBV-related ACLF, and our findings were confirmed to be a very valuable biomarker for predicting chronic liver disease severity. Further studies with larger patient groups and longer follow-up duration are required to verify our findings concerning the function of PWR in the inflammatory process of HBV-infected patients.

## Figures and Tables

**Table 1:** Clinical characteristics of studied subjects.

	Healthy controls (*n* = 97)	ACLF (*n* = 433)	*p*
Age (year)	40 ± 11.7	42 ± 11.44	0.123
Gender (male/female)	72/25	360/73	0.059
White blood cell count (×10^9^/L)	5.86 ± 1.53	7.72 ± 4.06	<0.001
Blood platelet (×10^9^/L)	228 ± 52.54	111 ± 64.55	<0.001
PWR	39.16 ± 9.80	14.03 ± 7.15	<0.001
International normalized ratio (INR)	0.98 ± 0.52	2.58 ± 1.09	<0.001
Alanine aminotransferase (U/L)	20 ± 12.8	361 ± 848.14	<0.001
Aspartate aminotransferase (U/L)	20 ± 5.6	204 ± 668.87	<0.001
Total bilirubin (*μ*mol/L)	13.23 ± 4.95	369.4 ± 165.51	<0.001
Albumin (g/L)	47.3 ± 2.23	31.7 ± 13.80	<0.001
HBV DNA level (log IU/ml)		4.87 ± 2.14	
Mortality	0	129	<0.001

**Table 2:** Analysis of factors associated with the PWR value in HBV-infected patients. Scatter diagram for correlation analysis.

Factor	PWR	
	Spearman *R*	*p*
PTA	−0.1076	0.025
TBIL	−0.1485	0.002
RBC	0.2348	<0.001
HB	0.1887	<0.001
MELD score	−0.1410	0.003
HBV DNA	0.2230	<0.001

**Table 3:** Independent predictors of mortality by Cox proportional hazard regression.

Predictor	HR	95% CI	*p*
PWR	0.660	0.438–0.996	0.048
Age	1.042	1.025–1.059	<0.001
MELD score	1.083	1.049–1.119	<0.001

Variables included in the analysis were age, hemoglobin, PWR, MELD score, and total protein. MELD, model for end-stage liver disease; PWR, PLT to WBC ratio.

**Table 4:** Clinical and laboratory characteristics among patients with different PWR values at admission.

	High group (PWR ≥ 9.0, *n* = 336)	Low group (PWR < 9.0, *n* = 97)	*p*
PWR	9.27 ± 2.87	20.75 ± 5.38	<0.001
Age (year)	42.14 ± 11.53	44.87 ± 11.01	0.039
Gender (male/female)	286/50	74/23	0.032
WBC (×10^9^/L)	8.23 ± 5.42	10.46 ± 4.60	<0.001
Hemoglobin (g/dL)	123.49 ± 23.50	111.68 ± 21.94	<0.001
PLT (×10^9^/L)	133.76 ± 64.40	68.66 ± 31.57	<0.001
ALT (U/L)	811.66 ± 895.56	197.79 ± 238.61	<0.001
Total bilirubin (*μ*mol/L)	365.73 ± 153.43	424.41 ± 196.74	0.008
Albumin (g/L)	32.69 ± 15.46	30.31 ± 4.52	0.135
INR	2.88 ± 1.12	2.81 ± 1.02	0.545
Creatinine (*μ*mol/L)	75.59 ± 48.20	95.14 ± 70.68	0.012
MELD score	26.60 ± 5.07	28.11 ± 6.76	0.043
HBV DNA (log IU/ml)	5.21 ± 2.15	4.23 ± 2.08	<0.001
Ascites, *n* (%)	217 (64.6%)	71 (73.2%)	0.071
Mortality, *n* (%)	89 (26.5%)	40 (41.2%)	0.004

Data were expressed as the mean ± SEM.

## Abbreviations

HBV: Hepatitis B virus
ACLF: Acute-on-chronic liver failure
CI: Confidence interval
ALT: Alanine aminotransferase
TBIL: Total bilirubin
PTA: Prothrombin time
INR: International normalized ratio.

## Supplementary Materials

Supplementary Materials Supplementary Figure 1: Kaplan-Meier survival curves of the ACLF patients categorized by the MELD scores. The curves showed different survival rates of patients with different MELD scores.
