# Supplementary material for: Low Platelet to White Blood Cell Ratio Indicates Poor Prognosis for Acute-On-Chronic Liver Failure
Source: Biomed Res Int. 2018 May 8;2018:7394904. doi: 10.1155/2018/7394904 (PMC5964479; doi:10.1155/2018/7394904)
Supplement: Supplementary Materials — Supplementary Figure 1: Kaplan-Meier survival curves of the ACLF patients categorized by the MELD scores. The curves showed different survival rates of patients with different MELD scores. [file 7394904.f1.docx]

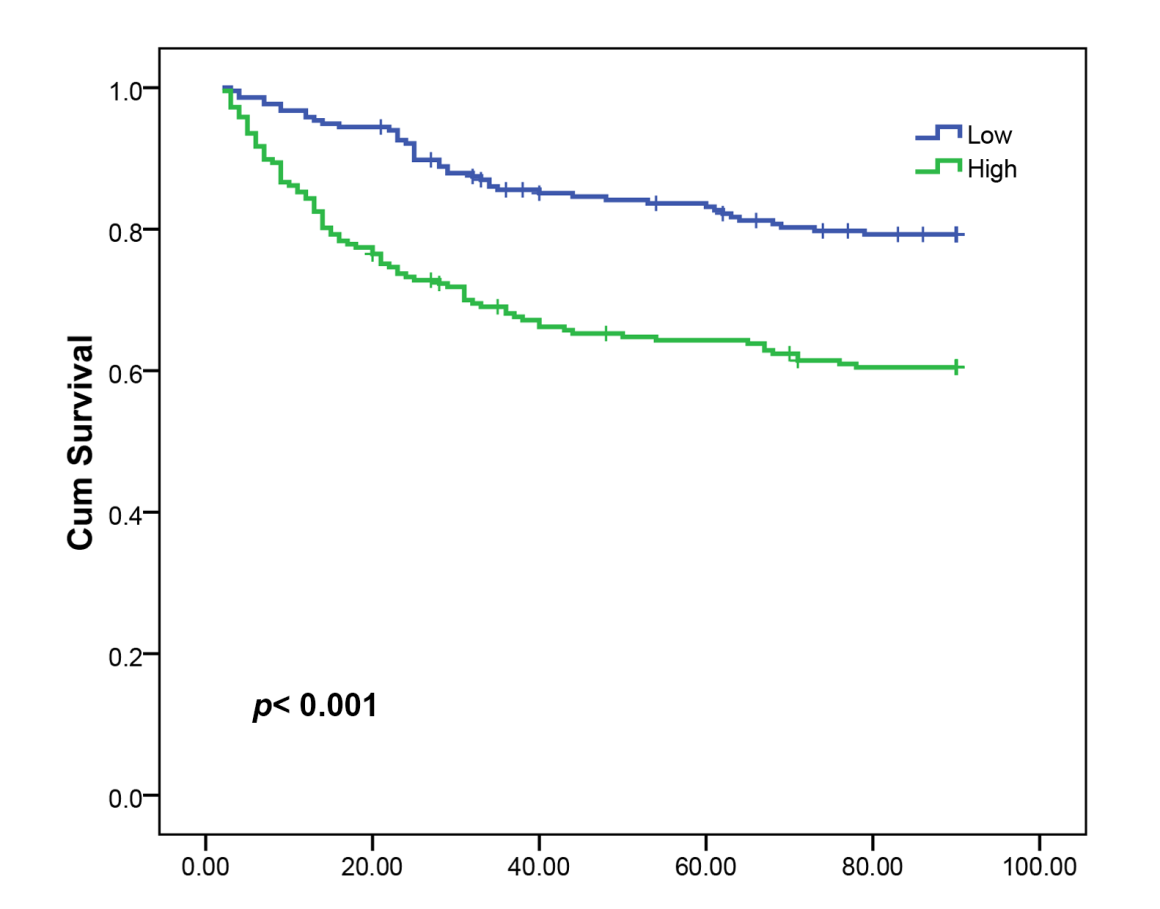


**Supplementary Figure 1.** Kaplan-Meier survival curves of the ACLF patients categorized by the MELD scores. The curves showed different survival rates of patients with different MELD scores.
